# Estimated frailty prevalence among Israeli elderly – results from a cross sectional national survey

**DOI:** 10.1186/s13584-018-0212-5

**Published:** 2018-04-12

**Authors:** Assaf Buch, Lital Keinan-Boker, Yitshal Berner, Eli Carmeli, Rebecca Goldsmith, Naftali Stern

**Affiliations:** 1 Institute of Endocrinology, Metabolism and Hypertension, Tel Aviv Sourasky Medical Center, Tel-Aviv, Israel; 2 The Sackler Faculty of Medicine, Tel-Aviv University, Tel-Aviv, Israel; 3 Robert H Smith Faculty of Agriculture, Food and Environment, The Hebrew University of Jerusalem, Rehovot, Israel; 4 School of Public Health, University of Haifa, Haifa, Israel; 5 Israel Center for Disease Control, Israel Ministry of Health, Ramat Gan, Israel; 6 Meir Medical Center, Kfar Saba, Israel; 7 The Department of Physical Therapy, University of Haifa, Haifa, Israel; 8 Nutrition Department, Ministry of Health, Jerusalem, Israel

**Keywords:** Elderly, Frailty, Israel, “MABAT Zahav”, Prevalence

## Abstract

**Background:**

Increasing longevity presents new social and medical challenges in developed countries. The prevalence of frailty is of interest because of its association with health prognosis and outcomes, but so far there is no single best diagnostic tool for this entity. Therefore, estimated prevalence of frailty in countries varies considerably and ranges between 5% and 58%. In Israel, the nation-wide prevalence of frailty in the elderly population is presently unknown. The objective of our study was to assess the rate of the frailty in elderly Israelis.

**Methods:**

A *post-hoc* analysis based on the results of a national Health and Nutrition Survey in Israeli elderly (MABAT Zahav). A non-direct model to estimate frailty was based on five components that were most similar to the common frailty assessment suggested by Morley et al. The frailty state was then reclassified according to different explanatory variables.

**Results:**

Data collected from 1619 subjects (F/M = 52.9/47.1%) with an average age of 74.6 years were analyzed. Estimated frailty prevalence in the elderly population was 4.9%. Frail people were more likely to have a lower income, be unemployed and have a lower education level. Frailty rates were higher in women, in Jews and in subjects more prone to low physical function.

**Conclusions:**

The estimated frailty prevalence in the Israeli elderly population, while relatively low, is comparable to some of the rates suggested in the literature. The factors associated with frailty in the Israeli population are in accordance with the existing literature. The suggested model may be helpful in identifying frailty in Israeli elderly.

## Background

Longevity has been increasing steadily. According to the Israeli Central Bureau of Statistics, life span in Israel rose by 8.7 years for men and 8.9 years for women in the last 4 decades. Life expectancy in 65-year old Israeli subjects is ~ 20 years, leaving an expanding and delayed time window for the evolution of the understudied loss of health and function [1]. In western countries, the proportion of people over age 60 is increasing faster than that of any other age group [2]. Frailty as a biologic syndrome is characterized by decreased reserve and resistance to stressors, leading to vulnerability to adverse outcomes [3]. Frailty is associated with an array of different conditions, such as lower cardiac function, hypertension and metabolic impairments (obesity and diabetes), arthritis [4–7] and is a risk factor for mortality, hospitalization, decreased function and falls [3, 8]. The detection of frailty however, with or without the presence of other co-morbidities, is heavily affected by differences in diagnostic methodologies [3, 9–14] and, as previously stated, “there is no single, generally, accepted clinical definition of frailty” [15]. However, in recent years attempts are made to reach consensus for frailty definition [16, 17]. Information on the approximate rate of frailty might be helpful to public initiatives aspiring to minimize its occurrence and constrain its social, economic and health costs in the face of a rapid rise in the elderly population.

Specialist comprehensive geriatric assessment (CGA) [18] is considered as a reference standard test for the identification and management of frailty in hospitalized subjects [19] and home assessment services [20]. However, since the CGA is time consuming and requires much expertise, multiple attempts by researchers and working groups have been made to reach consensus regarding a simple but accurate way to diagnose frailty. The diagnostic methods suggested included direct functional evaluation and single or multiple tests [10, 13, 14, 21–23], with some of these diagnostic criteria validated against the CGA and some against the Fried phenotype model [3]. Another simple and widely common frailty tool was introduced by Morley et al. [24] which is composed of different functional aspects (1. report of fatigue; 2. inability to climb one flight of stairs; 3. inability to walk one block; 4. > 5 illnesses; 5. weight loss >  5%). Moreover, in the diagnosis of frailty, factors such as polypharmacy [25, 26] or co-morbidity [27] (separately or combined), subjective health perception [25, 26, 28, 29], low physical activity performance [3, 26], weight loss [3], lower activities of daily living (ADL) or disabilities [3, 26–29], low cognitive score and mood disturbance/depression [8, 25, 27] have all been suggested as independent or interlinked components of frailty.

Given the variety of diagnostic approaches, it is hardly surprising that the estimated prevalence of frailty prevalence in elderly ranges between 5% to 58% [13]. National Surveys and studies performed on the frailty state may be limited because of the costs involving direct physical examination of patients and reliance on remote surveying techniques. In the “MABAT Zahav” survey, frailty state has not been evaluated [30] and no estimation of prevalence of frailty among Israeli elderly currently exists. The Israeli population is culturally diverse and composed of Jews, Muslims and Christians; encompassing natives and immigrants from different countries (Europe, America and others). Hence, the Israeli population may comprise an interesting model to assess frailty on the background of ethnicity variations. Although the first “MABAT Zahav” survey has not directly measured physical function, nor has it directly recorded some of the accepted criteria for frailty, this national survey in the elderly Israeli population (aged 65 and over) is highly informative and population representative. Our objective was to construct, for the first time, a *post-hoc* estimation model for the assessment of the prevalence of frailty which was applied on a database collected in elderly Israeli and included multiple aspects of health, functional, cognitive and mood status.

## Methods

### Population and sampling in the survey

The first cross-sectional MABAT Zahav survey is a National Health and Nutrition Survey of the elderly aged 65 and over in Israel, which was carried out over a-one-and-a-half-year period, between July 2005 and December 2006, by the Israel Center for Disease Control and the Nutrition Department of the Israel Ministry of Health. It was a national survey on the health and nutrition status of a random sample of elderly [fully described in [31]]. It included 1852 community dwelling elderly (1536 Jews; 316 Arabs, ≥65 yr), residing either in their own homes or in sheltered housing. Exclusion criteria included absence from the country or hospitalization for > 6 months; immigration to Israel after 31.12.2003; or significant cognitive impairment*.*

### Data collection

A personal face to face interview was conducted in the interviewees’ homes using a structured questionnaire. The full questionnaire included demographic details, questions on health status, lifestyle as well as descriptive details addressing the functional, cognitive and emotional state. Height, weight, body circumferences and blood pressure were measured. Examination of function was based on the Katz scale of ADL [32], which was an integral part of the questionnaire, and assessed the ability to dress, shower/bath, sit down and rise from a chair, eat and go to the bathroom. The maximum score was 15, with a score of 5 indicating “no functional limitations”, a score of 6–10 indicating “some functional limitations”, and a score of 11–15 indicating “several functional limitations”. Cognitive assessment was carried out using the Mini–Mental State Examination (MMSE) [33]. The maximum score was 30. The MMSE scores were adjusted for education and age as is routinely done, using the Crum tables. Cognitive impairment was defined based on the MMSE scores, which were dichotomized to determine cognitive decline (a score > 17 and < 24) and normal cognitive function (a score = 24+) [33, 34]. It should be clarified that subjects with MMSE scores 17 or lower were excluded from the analyses due to potential report bias. The assessment of mood disturbance (depression) was carried out using the 12 item General Health Questionnaire (GHQ) [35]. A score of < 4 indicated no mood disturbance; a score of 4–8 indicated mild disturbance whereas a score higher than 8 (up to 12) indicated significant disturbance. More information on the protocol is available elsewhere [36].

### Frailty estimation assessment

To get a closer insight of our objective we re-analyzed the first MABAT Zahav data [30] (*n* = 1852; age range 65–97). As explained earlier, we excluded subjects with potential report bias as represented by very low cognitive scores (MMSE score ≤ 17) (*n* = 16) [33, 34]. We built a non-direct model to estimate frailty based on 5 components that were most similar to the common frailty assessment tool suggested by Morley et al. [17] and the available literature (Table 1). The criteria were (% in sample): 1. Physical inactivity in the past year (38%); 2. Co-morbidity (≥ 4 diagnosed morbidities of the following: chronic renal failure, cardiac insufficiency, heart attack, stroke, Parkinson’s disease, asthma, hypertension, diabetes, osteoporosis and vision damage [cataract and/or glaucoma]) (10%); 3. Report on significant weight loss in the past year (> 3 kg) (6%); 4. Estimation of sarcopenia (low appendicular mass) using mid arm and calf circumferences (cut-offs of older adults’ national survey were imputed and adjusted by age group) (27%) [36]; 5. Low subjective health perception [26, 27, 29, 30] (self-assessment of personal health as same or worse compared to the previous year) (8%). (Table 2). There were no appropriate criteria obtained in MABAT survey to replace the resistance and the fatigue criteria suggested by Morley. Therefore, we used previously suggested variables that can define frailty, and which were also assessed in the survey (subjective health perception and appendicular circumferences). The model score was based on “yes = 1/no = 0” per each criterion, and ranged between 0 and 5. Being prefrail entailed having at least one criterion, while having three or more criteria determined a status of frailty as suggested in the widely used Fried phenotype model and in Morley 5 item frail scale [3, 24]. To evaluate the validity of the model we examined its power in detecting functional limitations using the accepted Katz criteria [32] (using any score >  5 representing the presence of some and several limitations) for the whole sample (excluding subjects with risk of report bias) and found fair validity (Area Under the Curve [AUC] 0.755; 95% CI 0.727–0.783). In total, 1619 MABAT participants had complete data for calculating their frailty state and were included in the current analyses (missing data on 217 subjects).

**Table 1:** Comparing our model for estimating frailty with the previous common and accepted model by Morley et al.

	Morley 5 frail scale model [17]		Our frailty model	
Component number	Criterion	Definition	Criterion used instead^1^	Definition
1	Aerobic	Cannot walk 1 block	Physical inactivity	Non- engagement of any intentional physical activity in the past year
2	Illnesses	> 5 diagnosed illnesses	Co-morbidity	≥ 4 diagnosed morbidities ^2^
3	Loss of weight	> 5% of the original weight in the past 6 months	Spontaneous weight loss	> 3 kg of the original weight in the past 1 year
4	Resistance	Question: “Cannot walk up 1 flight of stairs?”	Estimation of sarcopenia^1^	Low appendicular mass using mid arm and calf circumferences^3^
5	Fatigue	Question: “Are you fatigued?”	Low subjective health perception^1^	Self-assessment of personal health as same or worse compared to the previous year
	Defining robust	No positive scores	Defining robust	No positive scores
	Defining pre-frail	1–2 positive scores	Defining pre-frail	1–2 positive scores
	Defining frail	3 or greater positive scores	Defining frail	3 or greater positive scores

1- There were no appropriate criteria obtained in MABAT survey to replace the resistance and the fatigue criteria suggested by Morley. Therefore, we used previously suggested variables that can define frailty, and which were also assessed in the survey

2- We defined comorbidities as: ≥ 4 diagnosed morbidities of the following: chronic renal failure, cardiac insufficiency, heart attack, stroke, Parkinson, asthma, hypertension, diabetes, osteoporosis and vision damage [cataract and/or glaucoma]

3- Sarcopenia was estimated in the post-hoc analysis of MABAT survey using mid arm and calf circumferences (as proxy for appendicular mass). Cut-offs of older adults’ national survey were imputed and adjusted by age group. For males: a) aged 65–74: mid upper arm and calf circumferences < 29.9 cm and 26.6 cm, respectively; b) aged 75–84: mid upper arm and calf circumferences < 28.1 cm and 34.9 cm, respectively; c) aged ≥85: mid upper arm and calf circumferences < 27.7 cm and 33.7 cm, respectively. For females: a) aged 65–74: mid upper arm and calf circumferences < 31.8 cm and 37.7 cm, respectively; b) aged 75–84: mid upper arm and calf circumferences < 30.1 cm and 35.3 cm, respectively; c) aged ≥85: mid upper arm and calf circumferences < 26.9 cm and 34.6 cm, respectively [45]

**Table 2:** Fill out form for health practitioner to assess frailty likelihood - based on the frailty model using MABAT zahav data

This following form includes five components assessing several aspects of health related to the likelihood for frailty		
1	Over the past year, did the patient avoid regularly leisure time physical activity (10 min at least)?	Yes/No
2	Does the patient have ≥4 comorbidities ^1^	Yes/No
3	Does the patient have sarcopenia/ low appendicular (arms and legs) mass?^2^	Yes/No
4	Does the patient report on significant spontaneous weight loss in the past year (> 3 kg) ^3^	Yes/No
5	Does the patient report his/ her health condition as “not so good or bad”? and relatively health deterioration from the previous year?	Yes/No

1- In the post-hoc analysis of MABAT survey we defined comorbidities as: ≥ 4 diagnosed morbidities of the following: chronic renal failure, cardiac insufficiency, heart attack, stroke, Parkinson, asthma, hypertension, diabetes, osteoporosis and vision damage [cataract and/or glaucoma]

2- Sarcopenia was estimated in the post-hoc analysis of MABAT survey using mid arm and calf circumferences (as proxy for appendicular mass). Cut-offs of older adults’ national survey were imputed and adjusted by age group. For males: a) aged 65–74: mid upper arm and calf circumferences < 29.9 cm and 26.6 cm, respectively; b) aged 75–84: mid upper arm and calf circumferences < 28.1 cm and 34.9 cm, respectively; c) aged ≥85: mid upper arm and calf circumferences < 27.7 cm and 33.7 cm, respectively

For females: a) aged 65–74: mid upper arm and calf circumferences < 31.8 cm and 37.7 cm, respectively; b) aged 75–84: mid upper arm and calf circumferences < 30.1 cm and 35.3 cm, respectively; c) aged ≥85: mid upper arm and calf circumferences < 26.9 cm and 34.6 cm, respectively [45]

3- Originally according to Fried’s criteria a significant spontaneous weight loss was considered as > 4.5 kg, however, in MABAT survey the highest category was > 3 kg

Sum number of “yes” answers, if ≥1 and < 3 higher likelihood for pre-frailty, if ≥3, higher likelihood for frailty state, if =0 than no frailty state (robust)

Selected independent variables were chosen for comparison within the different strata of frailty: age, gender, BMI, smoking status, income, education, marital status, employment, cognitive and emotional function and the presence of obesity and other morbidities.

### Data analysis

Was performed by the SPSS software (Version 24.0). *P* values were considered statistically significant if lower than 0.05. Link of categorical variables was assessed by the Chi Square test, whereas ANOVA was chosen to examine the association of continuous variables with frailty strata. Jonckheere non-parametric trend test was used to test the hypothesis of a linear trend when variables were continuous across frailty strata.

## Results

### General results of the survey

Data of 1619 subjects were analyzed, of whom 52.9% were females. The average age of the total sample was 74.60 ± 6.12 years. Most participants were retired, unemployed or not engaged in volunteering activity in the 3 months preceding the survey (76.5%). The majority was married (64.6%) but a sizable fraction was widowed (29.3%). Religion or ethnic background was as follows: Jews (84.3%), Arab Muslims (8.1%), Arab Christians (5.7%), non-Arab Christian (1.7%) and Druze (0.2%) (Table 3).

**Table 3:** The Relationship between the likelihood of frailty and different variables – a univariate analysis

	Variable	Total population (*n* = 1619)^1^	Robust (*n* = 609; 37.6%)	Pre-frail (*n* = 930; 57.4%)	Frail (*n* = 80; 4.9%)	*P among groups†* **∗**
Gender	Females (%)	52.9	36.0	62.4	71.3	*< 0.0001* **∗**
Age	Age (mean ± SD)	74.60 ± 6.12	73.86 ± 5.64	74.92 ± 6.34	76.53 ± 6.43	*< 0.0001* **∗**
Marital status	Married/with partner (%)	64.6	74.2	59.4	53.8	*< 0.0001*
	Widowed (%)	27.7	19.4	32.3	36.3	
	Single (%)	2.5	1.3	3.4	1.3	
	Divorced (%)	4.4	4.4	4.0	8.8	
	Separated (%)	0.7	0.5	0.9	0.0	
Religion ethnicity	Jewish (%)	84.3	89.1	80.6	90.0	*< 0.0001*
	Arab Muslim (%)	8.1	4.0	11.2	3.8	
	Arab Christian (%)	5.7	4.0	6.9	5.0	
	Christian (not Arab) (%)	1.7	2.8	1.1	1.3	
	Druze (%)	0.2	0.2	0.2	0.0	
Smoking	Current smoker (%)	11.0	9.6	12.0	10.0	*0.026*
	Past smoker (%)	34.9	39.9	31.8	33.8	
	Non-smoker (%)	54.1	50.6	56.2	56.3	
Employed/volunteered in the last 3 months^a^	% Do not work/volunteer	76.5	67.1	81.1	95.0	*< 0.0001*
Education	Education years (*n* = 1612)	10.80 ± 5.17	12.34 ± 4.70	9.91 ± 5.20	9.35 ± 5.52	*< 0.0001* **∗**
Income^b^	≤1744 NIS (%)	3.6	0.7	5.1	8.8	*< 0.0001*
	1744+ NIS (%)	96.4	99.3	94.9	91.3	
Physical function	Katz ADL score (mean ± SD)	5.61 ± 1.51	5.18 ± .83	5.75 ± 1.65	7.25 ± 2.20	*< 0.0001* **∗**
	No functional limitations (Katz score < 6) (%)	81.04	93.27	76.88	36.25	*< 0.0001*
	Some functional limitations (score 6–10) (%)	16.68	6.24	20.22	55.00	
	Several functional limitations (score 11–15) (%)	2.22	0.33	2.90	8.75	
Mood (*n* = 1262)	GHQ score	6.45 ± 2.91	5.82 ± 2.98	6.75 ± 2.79	7.94 ± 2.60	*< 0.0001* **∗**
	Negligible disturbance (score 0–3) (%)	18.5	24.2	15.4	9.1	*< 0.0001*
	Moderate disturbance (score 4–8) (%)	61.8	63.5	62.3	43.9	
	Severe disturbance (score 9–12) (%)	19.7	12.3	22.3	47.0	
Cognitive function	Age-adjusted MMSE score	30.84 ± 3.48	30.87 ± 2.68	30.86 ± 3.84	30.24 ± 4.37	0.766
	Cognitive impairment (MMSE< 24)^c^	3.6	1.3	4.5	10.0	*< 0.0001*
Clinical and metabolic	BMI (mean ± SD; kg/m^2^)	29.18 ± 4.81 (*n* = 1514)	29.26 ± 4.08 (*n* = 595)	29.24 ± 5.17 (*n* = 857)	27.47 ± 5.72 (*n* = 62)	< 0.0001*
	Osteoporosis presence (%)	25.6	13.3	31.4	52.5	*< 0.0001* **∗**
	Physician diagnosed hypertension (%)	57.8	57.0	57.2	71.3	*0.044*
	Physician diagnosed diabetes (%)	28.2	21.2	30.1	58.8	*< 0.0001*

† = χ^2^ test for categorical variables and analysis of variance for continuous variables – among frailty categories (robust, pre-frail, frail)

**∗ =** *P* for trend across groups for continuous variables (*p* < 0.01) using Jonckheere non-parametric trend test

1- The original data set included 1852 subjects of whom 217 did not have sufficient data to assess frailty. Another 16 subjects had higher risk for cognitive dysfunction (MMSE< 17) which may have resulted in report bias. Therefore, they were also excluded

^a^Comparison of subjects who do not work to those who work

^b^Income lower than 1744 NIS/person/month was defined as the poverty line

^**c**^Cognitive impairment is considered as any MMSE score below 24 (but ≥17) and was compared to MMSE score ≥ 24

*Abbreviations*: *ADL* Activities of daily living, *BMI* Body mass index, *GHQ* general health questionnaire, *MMSE* Mini mental state examination, *SD* Standard deviation

### Frailty state in the entire surveyed population

Using the suggested cut-off levels for the frail and non-frail state, we estimated that the overall prevalence of robust state was 37.3%. The estimated prevalence of the pre-frail state (prevalence of at least one criterion) was 57.4% and 4.9% for the frail state (prevalence of at least 3 criteria). The frail subjects were more likely to be females than males (71.3% vs. 36.0% and 62.4% in the robust and pre-frail groups respectively; *p* < 0.0001 among all groups). Mean age slightly increased between groups- from 73.86 ± 5.64 in the robust group to 76.53 ± 6.43 in the frail group (*p* < 0.0001). The frail subjects were less educated as assessed by total years of education: 9.35 ± 5.52 vs. 12.34 ± 4.70 and 9.91 ± 5.20 in the robust and pre-frail groups respectively; *p* < 0.0001 among all groups). Further, the rate of unemployed/non-volunteering state was the highest in the frail group (95.0% vs. 67.1% in the robust and 81.1% pre-frail groups; *p* < 0.0001). The frail subjects’ income was dramatically lower than that of the robust group: 8.8% compared to 0.7%, respectively, had monthly incomes that were lower than the level defined as the poverty line in Israel (≤1744 NIS/person/month) (*p* < 0.0001 among all groups). Functional limitations were more prevalent in the frail and pre-frail groups, as also represented by an increase in the Katz score between the groups (from 5.18 ± .83 in the robust group to 7.25 ± 2.20 in the frail group; *p* < 0.0001) (Table 2).

## Discussion

In this analysis of data derived from a large cross-sectional national survey of the Israeli elderly population, we proposed a screening model for frailty. We used 5 variables formerly shown to be linked to frailty. We then compiled a model inclusive of these variables and applied it to estimate the prevalence of frail, pre-frail and non-frail subjects. Our model is composed of different components of frailty representing both subjective and objective assessment and covering a variety of health aspects as suggested elsewhere [22]. This model was based on the Frail Scale (by Morley et al.) which was previously shown to be one of the best predictive frailty tools for disability [37]. The criterion validity of our model was examined against the Katz’s ADL scoring and was found to be fair (AUC 0.755). In accordance with the existing literature, we included highly predictive frailty indicators for ADL disability in community-dwelling elderly such as low physical activity, recent non-intentional weight loss as well as lower extremity function (the latter presented by a proxy of extremity circumferences) [38]. We did not include, however, direct physical measurements which would expectedly be more valid and informative, but also more costly and less available.

The model presented herewith estimated that 4.9% of the entire older (≥65 yrs) Israeli population is frail. This rate is comparable with previous epidemiologic studies [3, 11]. In a cohort study conducted in the USA among 5317 men and women (≥65 years), frailty was assessed using the Fried’s criteria and estimated to affect 6.9% of the population [3]. The American population examined in this study [3] is ethnically diverse as is the Israeli population, a fact that may explain these similarities. Prevalence data are available also from 10 European countries using uniform criteria (questions on 5 parameters: weight loss, exhaustion, weakness, slowness and low activity). An overall prevalence of 17% (ranging between 5.8% in Switzerland and 27% in Spain) was disclosed [11]. A nationally representative survey conducted in 1992–1993 among 3107 respondents (age of 55–85 years) in the Netherlands estimated that 12% of the population was frail [39]. A survey of 7334 older adults (≥60 years) living in five large Latin American and Caribbean cities yielded a frailty prevalence rates of 21%–48%, using the Fried’s criteria [22]. When a similar screening process to the one shown here was implemented (using the FRAIL scale by Morley et al.) on 816 community Chinese elderly in Hong-Kong (≥65 yrs.), prevalence of pre-frailty and frailty were 52.4% and 12.5%, respectively [40]. Overall, using a meta-analysis of 21 cohorts (*n* = 61,500) on average, 10.7% of community-dwelling older persons are frail and another 41.6% are prefrail (range: 4.0% to 59.1%) [41]. This dominance of the pre-frail state (52.4%) was also found in our study (≈57%) and is mainly attributed to inactivity (38%).

In our study, the proportion of frailty observed is comparable to the lowest estimated rate in the European findings (5.8% in Switzerland) [11], but indeed slightly lower. These differences may be attributed to inter-country variations (socioeconomic gaps) and to the differences in the diagnostic tools utilized for the assessment of frailty. Additionally, our results, mostly based on self-reports, may have been also biased due to an underestimation of the interviewees of their true health status, either because of an attempt to satisfy the interviewer, or because of an over-optimistic approach: only 8% of the participants defined their current health status as bad and as being the same or worse than in the previous year.

The correlates for frailty shown in this study are supported by previous data. As expected, frail compared to non-frail subjects were more likely to be women, to be older and to earn lower incomes in accordance with the National Health and Aging Trends Study, a national longitudinal study of persons aged 65 and older conducted in the USA [42]. Lower educational level and lower proportion of having a partner in the frail subjects were observed in a survey from the Netherlands and also in this study [39]. Characteristics of other frail population using the FRAIL scale by Morley et al. are in accordance with our study showing that frail and pre-frail subjects were more likely to be women, to have cognitive impairment, to be unmarried [40]. This evidence shows that at most the population identified as frail in our report is consistent with characteristics of other frail populations, which supports our model’s validity and accuracy.

The model suggested may serve as a screening tool to identify subjects at higher risk for frailty. It has been previously shown in a meta-analysis of 31 studies that pre-frailty and frailty were both associated with higher risk for premature mortality, hospitalization and disability [43]. Therefore, simple tools to identify individuals at risk may assist primary physicians as well as health organizations to assess the magnitude of the problem and to apply preventive measures to defer the health sequels of frailty.

The model we developed is simple and apparently reproducible, as it may be easily utilized in observational studies. The scoring in the model is comprised of frailty-related variables previously used in other screening-diagnosing tools [3, 17]. It remains to be seen, however, whether or not this model might be useful to reflect or predict the natural prognosis of frailty state, as well as its progression under interventions. Furthermore, even if the current model performs well against Katz’s ADL, it may not perform well for “frailty” as proposed by the Fried criteria [3] or by Morley frail scale [24].

Another limitation of our model is that it is, indeed, a post hoc analysis of a survey which did not intend to assess frailty but rather addressed several variables associated with frailty. To circumvent this limitation, we used several and different representative variables, based on the scientific literature, to estimate the frail state and also compared our results with other studies. Although the estimated frailty rates in our study yielded comparable rates and correlates to previous reports which used a variety of different methodological approaches, our findings will require confirmation by alternative research routes. Finally, the data presented here is taken from a survey conducted over 10 years ago. With the rapidly increasing lifespan, as the population continues to age, frailty rate may change as well, such that the comparison of our analysis with past and future studies naturally requires awareness to this added complexity.

## Conclusions

In conclusion, this study is the first to estimate the prevalence rates of the frailty state in a representative sample of the elderly Israeli population. Rate of frailty in Israeli elderly was comparable to the lowest rates shown in the literature and was associated with known variables such as low socioeconomic status, female gender and lower functional and cognitive status.

The main health policy-related implications of the current study address several aspects. The first, for the primary physician, the study provides a simple, easily applicable screening tool for the identification of frailty proneness, a task that usually requires expertise or depends on relatively lengthy questionnaires. The possibility to correctly identify patients at risk for frailty in primary care settings serves (i) to better prioritize further consultations with less accessible specialists such as geriatric physicians; and (ii) to assist the primary physician’s in making decisions with respect to major surgery, cancer treatment, management of congestive heart failure, and even in predicting lower benefit of Influenza vaccination [44]. The second, the model may be used to easily assess the extent of frailty among the members of health care funds and help them plan, prioritize and allocate resources to address the needs of this high-risk group of patients. For example, targeting the appropriate population for long-term care insurance plans. Lastly, the tool can be useful in translational research and can be implemented for applied studies on elderly populations.
